# Liverpool Centre Inaugurated

**Published:** 1918-03-02

**Authors:** 


### THE COLLEGE OF NURSING.

### LIVERPOOL CENTRE INAUGURATED.

### Sir Arthur Stanley and the Critics.

### VISCOUNTESS COWDRAY'S SPEECH.

### An Opposition "Scene."

THE Liverpool Centre of the College of Nursing, Limited, is now an accomplished fact. The inauguration was auspiciously consummated at a large and influential meeting in the Council Chamber of the Town Hall on Friday, February 22. In the absence, through illness, of the Lord Mayor, the chair was occupied by Dr. Richard Caton (Deputy Lord Mayor), and amongst others present were the Viscountess Cowdray (Hon. Treasurer of the Nation's Fund for Nurses), the Hon. Sir Arthur Stanley, G.B.E., M.P. (Chairman of the Council of the College), Sir W. Scott Barrett, Miss E. M. Cummins (Matron, Royal Infirmary, Liverpool, and a member of the Council of the College), Miss Alison Garland, Miss Worsley (Children's Infirmary, Liverpool, and Hon. Secretary of the Liverpool Centre of the College of Nursing), Mr. A. H. Maxwell (Chairman of the Liverpool Children's Infirmary), Sir James Barr, Dr. E. W. Hope (Liverpool Medical Officer of Health), Mr. Thomas Woodsend (President of the Royal Southern Hospital, Liverpool), Dr. T. H. Bickerton, Alderman Muirhead, Alderman Crosthwaite, and Mr. J. Corbett Lowe.

**The Chairman** said that they all regretted very much that the Lord Mayor, in consequence of indisposition, was unable to take the chair that day. His Lordship's interest in the subject they were about to discuss was so great that his absence would no doubt be almost as great a matter of regret to him as it was to them. A great many letters had been received from persons who would have wished to be present, but who were unavoidably prevented. Amongst the writers of those letters were the Bishop of Liverpool (the Rt. Rev. Dr. Chavasse) and Mr. H. Wade Deacon (Chairman of the Liverpool Royal Infirmary). There were a number of ladies and gentlemen in that room who, like himself, had watched the progress of the great profession of nursing for a length of time—some of them for fifty years or more. The changes which had taken place in that time had been of the most astonishing character. He would like to say before he sat down that as Chairman of the West Lancashire Nursing Services' Association it had been his duty and pleasure to spend a great deal of his time in the hospitals ever since the war began. He wanted to say how magnificent had been the services of their trained nurses—how perfectly magnificent. Also let him say that a large body of V.A.D. nurses had assisted them.

**Sir Arthur Stanley** said he desired to echo what the Deputy Lord Mayor had said about their regret for the absence of the Lord Mayor owing to illness. He could not possibly have chosen a better substitute than he had in his Deputy. Dr. Caton was well known not only as one of the previous Lord Mayors, but also as one who had done an immense amount for the nurses and the nursing profession in that part of the world. There was a meeting in Liverpool a little time ago at which views were expressed not entirely in harmony with the College. He wanted to say this, and to make it perfectly clear from the beginning; it took two people to make a quarrel, and the College of Nursing was not going to be one. (Hear, hear.) The College started from a very small beginning. It grew up because those of them who only since the war had become intimately associated with nursing, felt that a body such as the College was absolutely necessary.

THE COLLEGE WORKS FOR UNITY.

All the way along, ever since the inception of the College, they had done their best to obtain unity of the nursing profession. They tried to get an agreed State Registration Bill; they practically got it, but then, for reasons into which he need not then go, negotiations were broken off for the moment. They then tried an amalgamation with the Royal British Nurses' Association, a body which had been in existence for a great many years, and had done a certain amount of work. At a large meeting that amalgamation was unanimously agreed by the Royal British Nurses' Association, but subsequently, for reasons known to themselves, they did not carry it out. He still believed that a large body of the members belonging to the Royal British Nurses' Association would wish to do what all members belonging to the College of Nursing wished to do, that was, to see a junction of the two bodies for the good of the nursing profession. In a good cause like that one could not be discouraged. As far as he had anything to do with the College of Nursing and those with him, they would work for the unity of the nursing profession and State Registration, and he had not the slightest doubt they would get both.

OBJECTS OF THE COLLEGE.

Nurses had got the vote, but the vote and Registration for Nurses would not be of the smallest use unless there was some strong association of nurses, such as the College of Nursing, in order that they should derive the utmost benefits that could be given under both. Let him, then, come to the aims of the College. He had seen it stated at times that the College was all an invention of the employers of nurses, who, in some way or other, it was alleged, wanted to get the nurses under their own dominion. He had even been called what he never knew before it was a crime to be, but it had been held up against him that as Hon. Treasurer of St. Thomas's Hospital, London, he was an employer of labour. (Laughter.) He would just briefly go through the objects for which the College of Nursing was formed, and the objects to which it still adhered. The first object was: "To organise the nursing profession." He could only say as an onlooker and as the Chairman of the Red Cross, and in that capacity probably the largest employer of nurses at this moment, except the Army, that the nursing profession needed organisation very badly. The next object of the College was: "To secure State Registration for the trained nurse." About that there could be no question. The third object was: "To make and maintain a Register of trained nurses, and by this means to protect the nurses and the public." One of the objects for which the College was formed was to prevent the adoption by unregistered, uncertificated people, and often by women of very undesirable character, of the appearance of a nurse, and to prevent them from being allowed to assume in any way either the name or the dress of a nurse.

PROTECTION FOR THE TRAINED NURSE.

The next object was: "To protect the interests of trained nurses." Well, now, that might seem a comparatively small thing at first, but he believed that it would become eventually a very important function of the College. There was no doubt about it, in these days when legal processes and everything connected with the law were excessively complicated and excessively expensive, any individual was rather at a disadvantage. That might specially be said of the nurse. One of the most essential things about a nurse was her character. After all, character was one of the things most easily attacked and at the same time one of the things most difficult to defend. At present, if a nurse were badly treated, say, at a private case, what remedy had she got? She could go to law if by any chance she were defrauded, for instance, of the fees which were justly due to her. She could go to law, but if an individual nurse went to law on her own account, she knew that it would be very expensive and that she would waste a great deal of her time; she was almost powerless. He wanted to have behind the nurses a strong corporation like the College of Nursing that would see that the nurses got their due rights. He had known cases himself where nurses had been thoroughly badly treated and had been able to secure no compensation—nothing at all. He thought that when they knew that there was behind the nurses the College of Nursing that was standing up for and supporting the rights of nurses, they would think twice before they tried to defraud any nurse of her just dues. (Applause.)

### A Uniform Standard of Training.

The next object was: "To raise and maintain the standard of training." Well, that was really the beginning, the origin of the College. The College really took its origin in the question of training. Having to get nurses for the Red Cross, and having got a very large number of nurses also for the military service, they found that the standard of proficiency varied enormously. There were some great hospitals, such as those in Liverpool, whose standard of training could be taken as absolutely perfect. They got nurses from the Royal Infirmary, Liverpool, or from any other of the great hospitals in the city, and if they had had a three years' certificate with the nurses from those hospitals they knew that they were getting the genuine article. On the other hand, nurses came up with certificates from other hospitals, quite good in themselves, but in some cases certainly not qualified to act as training-schools. In other cases they came up with certificates of training of less than three years, which, though they might be good sometimes, in the main were not. There was every possible difference and divergence in the various certificates with which they had to deal. The College took its origin in the desire of those connected with it, who were very largely matrons or people connected with the large training-schools, to obtain a uniform standard; that was to say, when a nurse had got a certificate from whatever training-school she had received it, it meant that she had gone through a certain curriculum and reached a certain stage of efficiency.

### "One Portal" with Uniform Curriculum.

The next object was: "To establish a uniform curriculum of training, and one-portal examination." The "one-portal examination" had been rather misunderstood. It had been felt in some places in the country that they intended that every nurse wherever she was being trained should go up to London and pass an examination there. Well, of course, that would be absolutely impossible and ridiculous. But it was not impossible to do as the great Universities had done, and to establish local examinations, all conducted from one centre, and all conducted in such a way as to make a general standard of efficiency for all those who passed those examinations. That was what they proposed to do; what was really largely the interest in the meeting that day was the fact that that centre in Liverpool was the first which he hoped would take up that particular branch of the work.

### Lectureships and Scholarships.

Another object of the College was: "To establish lectureships, scholarships, and in every way to promote the advancement of the nursing profession." What they found pretty universally was that once a nurse had served her period of probation, done her three years' work in the hospital and had obtained her certificate, there was very little inducement, and even very little possibility, for any of these women to continue their training, if they wished to do so, in any particular branch of the work. Once they became nurses, certificated nurses, they seemed to have reached the end of all their ambition, and they remained that for the rest of their lives. Well, now, there must obviously be among the large number of trained nurses in the country some who had got a special aptitude in special branches. They wanted to pick out those nurses and to give them the possibility of continuing their studies in those particular branches—going abroad if necessary, and studying the methods abroad, going to America, where nursing education was very far advanced, and getting the very best out of every other country, and not only remaining in our own.

### Nurses Must be Better Paid.

He took it that in "the advancement of the nursing profession" might very well be included the question of the advancement of the pay of the nursing profession. (Hear, hear.) But as there might be some governors of hospitals there—he was one himself—he would not go any further into that question except to say, at the risk of losing his position as treasurer of St. Thomas's Hospital, that he thought that nurses were underpaid. (Applause.) He gathered from that applause that some of the nurses agreed.

### Local Clubs for College Members.

There were other things that could be done by the College, and one to which he attached a good deal of importance was that the College itself in London and the various centres in the big towns of the country should really be made the local centres for the nurses, places where they could go, meet one another, and meet their friends; places more like clubs, where they could have reading-rooms, where they could go in the leisure that they did not very often get, but where they could spend the amount of leisure they did get away from the atmosphere of the hospital. If he could judge of their feelings about a hospital from his own feelings about the Red Cross, they would be very glad to get away from it sometimes. Those were roughly and briefly the aims of the College. He did not think that any of those present were likely to think that they had been too little ambitious in the programme which they set out to attain.

### The Opposition Cry.

Well, then, as to the constitution of the College. There had been a good many wild statements made about it. He had seen it stated among the opponents, who he thought were comparatively few in number, but who certainly made themselves heard, that the constitution of the College was tyrannical and undemocratic. He always wondered when he read those statements, or heard them made, whether the people who made them had ever read or taken the trouble to find out anything about the constitution of the College at all, because if anything were the reverse of tyrannical, and if anything was democratic, it was actually the constitution of the College of Nursing. According to the people who made those statements the tyranny was supposed to come from the laymen who had founded the College, in order to enable them to tyrannise over the nursing profession. (Laughter.) They were told that there were a large number of laymen. They were obliged at the beginning to have seven laymen who had to sign the application to the Board of Trade, under which they were incorporated. Those seven gentlemen were very estimable voluntary workers in the Red Cross offices who were kind enough to sign the document without which formal application to the Board of Trade for registration could not be made. He thought he could answer for it that not one of them, since he signed that formal document, had expressed the slightest wish to have anything more to do with the College of Nursing. They were not likely, therefore, to interfere in any other way. Apart from those laymen he thought there were three others in the College of Nursing—one, he thought, in Scotland, one in Ireland, he was not sure about that, but all he did know was that he (Sir Arthur Stanley) was the third. His position on the Council of the College of Nursing was not exactly one of tyranny; if there was any tyranny at all, as Miss Cummins could corroborate, it was entirely the other way. He spent his life in trying to get on to the Register nurses who he thought ought to be on it, because as a mere man he was sorry for them, and he was invariably turned down by the College of Nursing. (Laughter.) Whenever he brought forward a proposal it was most carefully considered by the Council of the College of Nursing, who were all nurses practically, or doctors, but very few doctors. He could honestly say that his proposals were subjected to more rigorous criticism than any other proposals that were brought forward. It had also been pointed out more than once that he had to come up for election very soon, and that the nurses would have an opportunity of turning him off the Council altogether. He asked those who said that the management of the College of Nursing was tyrannical and undemocratic to remember that out of a membership now of over 8,000, only ten laymen were in the College of Nursing. He did not know how many doctors there were, but it was a very small number; he was well within the mark in saying that out of 8,000 members of the College of Nursing, at least 7,900 were actual nurses themselves.

### An Elected Council.

At first, of course, they had to have a nominated Council; they could not possibly get a Council elected before they had the people to elect them. They took for the nominated Council the shortest possible period—something, he thought, under two years. That period came to an end next April, when one-third of the nominated Council would resign. It would then be up to the nurse-members of the College to elect whoever they pleased in the place of those retiring members. Next year the second third of the nominated Council retired, and the year after the remaining third. In two years from now the whole of the Council of the College, without exception, would all be elected by the nurses themselves. They could not possibly have anything more democratic than that. He would tell them that he was responsible really for it being so democratic. When he proposed that it should be made elective as soon as possible, some of the nurses on the Council came to him and said that they thought that was rather dangerous, and that for the first few years at least it would be very much better if they had some nominated members. His reply was that if the nurses were worthy to have a College at all they were worthy to govern it; and that was his opinion now. (Hear, hear.) He felt perfectly certain that the members of the Council who would be elected by the members of the College next April or May would really represent the nurses, and would do everything they possibly could to uphold the standard which had already been set by the College. That was roughly the constitution of the College.

### The Period of Grace.

He would like to add something else. It had been said that they had admitted, or that they wanted to admit, V.A.D. nurses who had not had the full three years' training. That was absolutely untrue. The whole basis on which the College was founded was a Register of properly trained three years' certificated nurses. But certain arrangements had been made to provide for a "period of grace." The nurses, for instance, in a hospital like the London Hospital, who actually did four years' training, but did only two years in the hospital wards, or at any rate some of them—that was purely a temporary arrangement for what they called a "period of grace." Obviously, it would be unfair for the College of Nursing to come along now and by any arbitrary rules make it impossible for a woman who had looked upon herself quite fairly as a certificated nurse, to go on and earn her living. There must be a "period of grace," during which they might admit those who were properly certificated but who might for some technical reason or other not come up to the standard required. One case which had come before them lately was that of a hospital which had not got a resident medical officer. The stipulations of the College of Nursing were that the hospital, if it were to be a recognised training-school, must have a resident medical officer. Obviously, that was a rule that must be relaxed at a time like this, when the demands upon medical men for the Army were so great, and when it was not possible always for some of the smaller hospitals to have a resident medical officer. But in every case where they had had to make an exception, and admit a hospital to be a recognised training-school where there was not a resident medical officer, the hospital authorities had undertaken that as soon as the war was over they would have a resident medical officer in that hospital. The College now having been constituted, he was glad to tell them that they were getting on very well with their membership. He had had for the last few weeks to say that it was nearly 8,000. They had a meeting of the Council on the previous day when they passed 200 more nurses, and the membership was now over 8,000 nurses. (Hear, hear.) Moreover, their money was coming in all right too, which mattered a good deal.

### Great Nursing Centres.

The College having been constituted, the one great thing was to get the interest of various great nursing centres such as Liverpool, Manchester, Birmingham, Leeds, and others. They had already got very satisfactory branches started in both Scotland and Ireland. He was personally anxious, coming as he did from that part of the country, that Liverpool should be the first centre. He was proud to think that Liverpool was the first centre of the College of Nursing. It was right that Liverpool should be the first centre. Thanks to the efforts of Miss Cummins, Mr. Wade Deacon, Dr. Hope, and others, Liverpool was the first centre. (Hear, hear.) That meeting that day had been called to ratify that fact, and to ask them to sanction it with their approval.

### The Necessity for Money.

There was one thing certain, and that was that none of that work could possibly be carried on without funds. They had to look round for funds. There was a body known as "The British Women's Hospital." It was a wonderful association of ladies, originally all connected with the Stage, who had banded themselves together before the war, he thought it was to get the vote, but they did not say much about that now, because they had got it. (Laughter, and hear, hear.) Since the war began, however, they had engaged themselves entirely in works of mercy. They had raised a very large sum of money for the Scottish Women's Hospital, which had gone to Serbia and elsewhere, and had done such glorious work—work that was expressly connected with the name of Dr. Elsie Inglis. They had raised a vast sum of money, over £150,000, for the building of a Home for the Paralysed, which they hoped to erect on the site of the "Star and Garter." It was obvious that women who could work like that in the cause of the wounded men were also the right women to work in the cause of the nurses. They could not possibly have gone to better people, and he would say this with absolute sincerity, that they could not possibly have gone to people who would have been more kind. They set out to raise one joint Fund, a "Nation's Fund for Nurses," divided into two parts, one an endowment for the College, and the other a Benefit Fund to help nurses in time of sickness or distress. He would not say anything about those, but he could not help giving them one quotation. There was a lady who was the Editor of the *British Journal of Nursing*, well known to a good many of them, who had been a pioneer in nursing work for a great many years, but unfortunately, for some reason that he had never been able to discover, she did not like the College of Nursing, although he thought they were carrying out actually the objects which she herself wanted. She had said that this appeal for funds was pauperising the nursing profession, that they were holding up the nurses as an object of charity, and that they had got no right to do it. Well, as regarded the Benefit Fund, he could only say that if it were holding the nurses up to charity to ask the nation to subscribe a Fund which would help them in sickness, or old age, or distress—if that were a cause for reproof, well then the same thing applied to the British Red Cross in connection with the soldiers. He did not think anyone would hold that they were degrading the soldier by raising over £9,000,000 to help him. The country had felt that they were only paying a just tribute to his deeds. He (Sir Arthur Stanley) thought the same thing with regard to the Nation's Fund for Nurses. This was

### What Mrs. Bedford Fenwick Said.

"Educational advantages for nurses mean a direct gain to the public, and I think you will agree with me that it is not just that the whole financial burden of the further advance of nursing should be entirely borne by nurses themselves. In other and richer professions the public take their share in financial support. Witness the magnificent universities, the endowed professional chairs, the medical colleges, public libraries, and numerous organisations which afford opportunities of study to different sections of workers, resulting in the ultimate benefit of the community at large, but owing their existence to the munificence of a comparatively few public-spirited persons.

"I claim that the time has come when nurses need their educational centres, their endowed colleges, their chairs of nursing, their university degrees, and State Registration, and the present seems the psychological moment to come to the public, not as strangers, but as professional workers, known and trusted through the length and breadth of the land. and to urge that, as nurses pour out on its behalf a skill and devotion for which gold is no real recompense, the public shall now prove its appreciation and interest in the noble work of nursing by giving something of its wealth to peace nursing education and the status of the trained nurse on a strong financial basis.

"Is it too much to hope that the wealthy will come forward and found colleges of nursing—colleges in which the teaching power of the profession would be focussed and centred, which would put the apex on our training course, and by improving the standard of nursing the sick confer a real and lasting benefit on humanity at large?"

Sir Arthur Stanley, continuing, said that was his final word, because he could not possibly put the case better. He wanted them to feel, especially the nurses there, that in helping to establish that Liverpool Centre that day they were carrying on the great work which Florence Nightingale began in the Crimea; they were doing all in their power to raise and maintain the standard of nursing; and they were doing all in their power not only to help forward themselves, and to look after their own interests, but to further the interests of all those gallant women who belonged to the finest profession in the whole world. (Applause.)

British Women's Hospital Committee.

**The Viscountess Cowdray:** Sir Arthur Stanley had kindly referred to the British Women's Hospital Committee, and she thought it might interest them a little perhaps to know what that body really was. It was originally the Actresses' Franchise League. When the war began they were, of course, anxious to do war work instead of work for the franchise. As to the work in connection with the British Women's Hospital Committee, it was originally given that name because it was intended to be a hospital in France, but they were told it was not required in France. They struggled to do their work with the greatest possible economy. They did not pay anything that they could possibly help; everything was voluntary. The members gave all the time they could, and all the talent they could, and when they had not sufficient time and talent in their committee-room, they went outside and claimed them from their friends. The other day in London they had their first meeting. It was a comparatively small meeting, but they got in £1,600 in the afternoon from that meeting. In connection with obtaining that £1,600, she did not think they had £6 to pay in the way of expenses. She thought that would speak well for the management. The public could rest assured that whatever money was put into the Nation's Fund for nurses went direct to the work. Everything else was usually done by voluntary work. For instance, the magnificent poster that they saw exhibited in that room was given to them by Captain Spencer Price, one of their best artists. He had a fortnight's holiday from the trenches, and he spent that fortnight's holiday in doing that poster. They paid him nothing for it. Then they had a splendid cartoon in *Punch* a fortnight ago, done by Mr. Partridge, and very delightful words recommending the work to the public. They paid nothing for that either. And so they went on with the greatest possible economy. They struggled to do as they did in the "Star and Garter." She believed, with all due deference to Sir Arthur Stanley, that he would be still prouder of the Red Cross, of which he was chairman, if he could do the work with the same economy. And so would many other large charities. The work for the "Star and Garter" never cost them more than 2½ per cent. to 5 per cent. Any business man in Liverpool would assure them that that was good management. She did not think she had anything more to say to them. She would only ask their personal help—the best they could give for the best possible work. The College was on democratic lines, and it was left entirely with the nurses to unite and help themselves. (Applause.)

"Once a Nurse Always a Nurse."

**Miss E. M. Cummins** (Matron of the Royal Infirmary, Liverpool, and a member of the Council of the College of Nursing) said she would speak as a nurse to the nurses present, because she was a fully-trained nurse, and had spent the whole of her professional life in hospitals. Therefore she knew all the aims, and sorrows, and all other phases of the nurses' life. When the College of Nursing was talked of, sometimes one heard one or two objections raised. One objection had been, "Why take up such a big problem as this in the middle of the war?" That was a thing that appealed to her in the beginning; she felt that they had so many of their profession abroad, and scattered everywhere, that they ought to have their opinion on the subject; but the war had gone on, and unless they were, like Mr. Micawber, waiting for something to turn up, she felt they ought to make a beginning some day. So with very great pleasure she went on the Council of the College of Nursing when Sir Arthur Stanley asked her to do so. Another objection was that there were so many matrons of hospitals on the Council. She had yet to learn that when a nurse became a matron she ceased to be a nurse. From her experience of nursing, she could say that she did not think that the fact of being a matron could ever eradicate the fact of being a nurse. Did nurses think that because they might become matrons some day, they would forget the days of their probationership? She had not. Further, it was also said that matrons were employers of labour. She did not know how that could be, when she was an employee herself. She did not look upon herself as an employer; she looked upon herself as a nurse, and always would do so. Then as to organisation, some people were rather dubious about organisation, and said, "Well, have not the hospitals been organised all these years, and the nursing profession been organised?" That was altogether a different thing from the nurse's education. The hospitals had been organised. She thought nearly every hospital before the war had pledged itself to provide so many nurses. Speaking for her own hospital, within twenty-four hours of the war breaking out they had redeemed that pledge, and the nurses for the Navy and the Army had left the hospital. She was sure that other hospitals did the same. The lack of organisation, so far as nurses were concerned, did not touch the emergency in which they found themselves.

Better Organisation, Better Pay, and Shorter Hours.

Of course, the profession was unorganised, she granted; it had tried to organise itself for years and years, but it had never achieved it until the present moment, because there did not appear to have been such an interest taken in organisation amongst nurses themselves until the College came along. Of course, there had been so much disagreement that they never came upon some method which appealed to all sides. The nurses had had their own hospitals; they had been able to go to their matron, or the sisters who worked above them, to ask advice; but still there were many nurses in the world who had no hospitals, and had no one to go to for advice. Therefore, they hoped the College would be the means of helping the nurses in difficulties in the future. There were many things which needed improvement. She hoped that in the future better pay would be forthcoming; and also shorter hours. The College of Nursing would help in that direction. This was not the time, of course, to speak of shorter hours, because all the hospitals were working with short staffs; but she hoped there would be a time when nurses would be able to study for examinations. The nurses were the poorest paid profession in the world.

Personality before Education.

Apropos of education for the nurses, she said she was a great believer in it. Ever since she had been a matron in a hospital she had tried to attract educated women to her as much as she could, and also to the hospital. But they must not think that there had been no education at all inside the hospitals. Outside people might think, "Have all these years gone by, and has there been no education at all inside the hospitals?" In reply to that she would say there had been a good deal of education. Speaking for her own hospital, it was systematically done. Their honorary medical staff gave lectures to and coached the nurses. It was obvious that that could not be done in the small training schools, and that was where the examinations of the College would help. Education, however, must not take the place of personality. (Hear, hear.) When they put education and other things before personality, they forgot the ideals of Florence Nightingale altogether. Liverpool should be the first centre of the College of Nursing. Liverpool was an ambitious city, and she was an ambitious woman. She knew London pretty well, and she knew it was rather apt to think it should govern the provinces. She did not think that Liverpool was inclined to be governed by London in any way. (Hear, hear.) As she was on the Council, she looked upon herself as a kind of link between Liverpool and London. She could not say very much about what the centre was to be. She hoped that the College would not always be on paper only. She hoped they would have a building in Liverpool some day, with a library, medical and otherwise, and so on; in fact, a centre in Liverpool which London might perhaps consider a rival. When the centre was really started, there would be a sort of club to which nurses could go for advice. They hoped to have meetings for them to come to, and to arrange post-graduate lectures for nurses. Nurses could do their little bit both in helping themselves and in getting other people to help as well. They would have a centre some day in Liverpool of which they would all be proud. (Applause.)

The Function of the College.

**Miss Alison Garland** said she could assure them that she was safe in prophesying that the workers would have to be trained to the highest state of efficiency, and have to be well organised, if they were going to bring back order out of the chaos of war. No profession needed it more than the nursing profession, and it was for that purpose that the brilliant idea came to Sir Arthur Stanley to have a College of Nursing. It was not going to displace existing societies. The whole aim of the College was to co-ordinate them all, and if they would not come in it would not be the fault of the College, that was going out of its way—and would go out of its way—to try to bring all the various societies together, so that they might stand as one great profession before the country. The Royal Charter they wanted was State Registration. For many years that had been advocated, and if they had only been in thorough earnest, and taken the necessary steps—which they might have taken—they might have had it long ago. They wanted somebody to back up their interests at headquarters. They hoped the College would take up the point for them. Adverting to the appeal for the Nation's Nurses' Fund, she denied that there was any charity in it at all. It sometimes happened that, through no fault of their own, nurses fell upon bad times. There were two matrons in that room who, in different workhouse infirmaries, had nursed Nightingale nurses—some of those splendid women who came forward at the request of Florence Nightingale.

This Should Not be.

Not only that, about five miles from where they were meeting that day, there was a workhouse infirmary where only recently she saw a nurse, who was only twenty-nine years of age, who, after being for two or three years in a hospital for tuberculosis, had taken the disease herself. The public authority gave her a sum of money to which they thought she was entitled. She had long since spent that money, and she had now come to the workhouse infirmary ward, where she had been fortunate enough to meet with a very kind master and matron, who were trying to make up by their kindness and attention what she lacked in the sanatorium. They did not want pensions for women who were in good health, but only for women who had fallen upon adversity.

Help Through the College.

The College of Nursing would not only help them financially and materially, but it would raise the ideal for all nurses. They wanted to attract to the profession of nursing clever and capable women, and the College of Nursing would help them to do that. When they came to have reconstruction in the new order of things after the war, they would want all the experience of the nurses to help them, and they could get it through the College. They were more in need of organisation, and of a College, than any other body of women she could remember, because nurses had no votes as a rule, inasmuch as they had no husbands or houses. They felt that the College was going to hold up to the nurses ideals. It was going to attract the capable, the efficient, and the energetic woman. It was no idle sentiment to keep that ideal before the nurses, because "Where there is no vision the people perish." (Applause.)

The Resolution of Thanks.

**Dr. E. W. Hope** (Medical Officer of Health for Liverpool) moved a vote of thanks to the speakers and the chairman. Personally he had not the remotest doubt that in a reasonable time a very large proportion of the entire body of nurses would have joined the College. To him the advantages were perfectly obvious. It seemed to him to supply a real need. While they did not wish to reproach themselves with delays, they could but feel that they had been trading upon the missionary spirit of the nursing profession for too long a time. The time had now arrived when great and tangible benefits were within their reach.

**Sir James Barr,** M.D., F.R.C.P., in seconding the proposition, said he had long looked to the nursing profession, and he had always been an admirer of that profession. He thought registration in the first place, and a higher standard of pay in the second place, were most essential for nurses. (Applause.)

The vote of thanks was put to the meeting and very cordially passed. Sir Arthur Stanley replied on behalf of himself and the other speakers. (*See also p.* 464, *column* 1.)

Why the Public have lost Patience.

This seemed to have brought the proceedings to a close, and the Chairman was on the point of leaving the chair when he remarked, "I have been asked if any questions can be put by anyone. I think not. The meeting is at an end."

**Miss Macdonald** (Secretary of the Royal British Nurses' Association) at this point rose at the back of the Council Chamber and said, "I had a definite pledge that questions could be asked to-day. I came up here from London at great sacrifice of time, and now you say I cannot ask any questions."

**The Chairman:** I am afraid we cannot have any questions. I must rule against them absolutely. The meeting is now at an end.

Dr. Caton then left the chair, and the audience rose for the purpose of dispersing. The voice of a lady was heard loudly to declare, "I protest against a broken pledge. I am a British woman, and like fair play. Again I protest against a broken pledge."

Two ladies, who gave their names as Miss Beatrice Kent, of the Nurses' Protection Committee, and Miss Margaret Breay, sub-editor of the *British Journal of Nursing*, afterwards visited the press table and requested that they should be allowed to protest against the decision of the chairman not to allow questions to be asked. It may be mentioned that in the notice sent out beforehand, explaining the objects of the meeting, it was expressly stated that Lady Cowdray could not reply to questions because she was giving the fullest possible information in her speech.

[Subsequently to the meeting two donations of £100 each, from Dr. T. H. Bickerton and Mr. G. Parker, the father of a trained nurse, were handed to Miss Cummins on behalf of the Liverpool Centre.]

